# Supplementary material for: Adverse Events to Comirnaty Vaccine Are Linked to Sex, Age and BMI: Should We Consider Reducing the Dose for Females?
Source: Vaccines (Basel). 2023 Feb 22;11(3):505. doi: 10.3390/vaccines11030505 (PMC10055873; doi:10.3390/vaccines11030505)
Supplement: Supplementary file 1 [file vaccines-11-00505-s001.zip › vaccines-2198945-supplementary.pdf]

Supplementary data. Questionnaire.

- Name
- Surname
- Sex (Female/Male)
- Age
- Weight
- Height
- In which facility do you work?
- COVID-19 vaccine administration dose (first or second dose)
- Date of first dose of COVID-19 vaccine
- Date of second dose of COVID-19 vaccine
- Did you have a suspected adverse reaction to COVID-19 vaccine? (Yes/No)
- Did the first reaction occur on the day of vaccination? (Yes/No)
- Onset date of first symptoms
- Date symptoms ended (if finished)
- Have you been to the emergency room because of this reaction? (Yes/No)
- Which of these symptoms have you had? Please, indicate the duration of each symptom:

  1 day, 2-3 days, 4-7 days, 7-14 days, 14-21 days.

  - Local reactions at the site of injections)
  - Fatigue
  - Headache
  - Muscle pain
  - Joint pain
  - Fever (TC <38°)
  - Fever (TC >=38°)
  - Swelling and soreness of axillary lymph nodes (on the vaccination side)
  - Chills
  - Skin rash (localized)
  - Diffuse rash
  - Anxiety/panic attack
  - Feeling of fainting
  - Loss of consciousness
  - Sudden abdominal pain
  - Insomnia
  - Diarrhea
  - Nausea
  - Vomiting
  - Swelling of the face

- Swelling of the tongue or the throat
- Transient facial paralysis
- Hypotension (sudden drop in blood pressure)
- Sweating
- Tachycardia (fast heart rate)
- Oppression or chest pain
- Shortness of breath
- Other

- If you checked "Other" specify,
- If you have had a fever report the maximum body temperature recorded
- To date the reaction is:
  - fully resolved,
  - resolved but with some aftereffects,
  - is getting better,
  - has remained unchanged/worsened,
  - I don’t know
- Have you had other vaccinations in the 4 weeks prior to receiving the COVID-19 vaccine dose? If yes, which vaccination?
- Have you ever had a reaction after receiving a vaccine? If yes, which ones?
- Have you had COVID-19? (YES/NO)
- Have you had any symptoms? (YES/NO)
- Did you have to take medication to relieve the symptoms? (YES/NO)
- Do you have any other observations you wish to report?
